# Analysis of risk factors for medication-overuse headache relapse: a clinic-based study in China

**DOI:** 10.1186/s12883-015-0422-1

**Published:** 2015-09-17

**Authors:** Zhihui Yan, Yuan Chen, Chunfu Chen, Congcong Li, Xiaojun Diao

**Affiliations:** Department of Neurology, Provincial Hospital Affiliated to Shandong University, 324 Jingwu Road, Jinan, Shandong 250021 China; Department of Neurology, Yantaishan Hospital, Yantai, Shandong 264001 China

**Keywords:** Medication-overuse headache, Relapse, Risk factors, Non-conditional multivariate stepwise logistic regression

## Abstract

**Background:**

Medication overuse headache (MOH) is the third most prevalent headache type after migraine and tension-type headache. A large number of studies on the long-term prognosis have shown that MOH has a high relapse rate after treatment. Although MOH relapse-related risk factors have been reported, no related research has been performed in China. Therefore, the purpose of this study was to analyze and evaluate the risk factors for MOH relapse in China.

**Methods:**

Eighty-six out-patients of Shandong Provincial Hospital who were initially diagnosed with MOH, and who had successful withdrawal treatment within 2 months, were chosen from March 2012 to July 2013. All subjects were followed up by the investigators of this study. Of the 86 subjects, 27 who had relapsed were compared with 59 who had not relapsed (i.e. the controls). Based on a standardized questionnaire, a database was created (with Microsoft Excel 2010). The data, which included 38 indexes, were analyzed by univariate analysis with chi-square test, Fisher’s exact test, *t*-test, or paired rank test. The statistically correlated (*P* < 0.05) variables were chosen as the independent variables, thereby enabling the calculation of the non-conditional multivariate stepwise logistic regression.

**Results:**

The independent risk factors for medication-overuse headache relapse were determined as headache frequency before drug withdrawal, duration of primary headache, and headache frequency after drug withdrawal.

**Conclusion:**

Headache frequency before drug withdrawal, duration of primary headache, and headache frequency after drug withdrawal may be the independent risk factors for MOH relapse in China.

## Background

Medication overuse headache (MOH) refers to headache occurring on 15 or more days per month and developing as a consequence of regular overuse of acute or symptomatic headache medication (on at least 10 or 15 days per month, depending on the medication) for more than 3 months. MOH usually resolves after the overuse has stopped [1]. MOH usually occurs in patients who have a history of primary headache, especially in patients with previous history or family history of migraine and in patients with frequent headache [2].

Recent studies have shown that MOH is the third most prevalent headache type, coming after migraine and tension-type headache [3]. These studies have also revealed an age-related involvement of MOH, even during childhood [4]. There is no large-scale epidemiological data on MOH in China. Dong [5] retrospectively analyzed the clinical features of 240 patients with MOH and discovered only two patients (0.8 %) had previously been given the diagnosis of MOH. Furthermore, the median time to diagnosis after the estimated onset of the disorder was 4.0 years. Therefore, MOH has rarely been diagnosed in China. The goal of MOH treatment is to withdraw the overuse of medication, reduce the extent and frequency of headache attacks, and prevent relapse [6]. Many studies consider the success criteria for MOH withdrawal as an improvement in MOH relapse. A large number of studies on the long-term prognosis of MOH showed that this disorder has a high relapse rate after treatment. In the first year after withdrawal, the relapse rate is the highest—between 22 and 44 % [7]. Although MOH relapse-related risk factors have been reported, no related research has been performed in China. Therefore, the current study aimed to investigate the risk factors for MOH relapse in China.

## Methods

### Research objective

*Case Source*: Eighty-six patients visiting headache specialists in the out-patient clinic of Shandong Provincial Hospital between March 2012 and July 2013, diagnosed with MOH for the first time, and with successful completion of the withdrawal treatment after 2 months were invited to answer a questionnaire. They were then monitored for 7.5 months. Patients with relapse were assigned to the case group, and those without relapse were assigned to the control group. The study was approved by the Ethic Committee of Shandong Provincial Hospital, and written inform consent was obtained from the participants after they became acquainted with all the procedures (reference number: 20140321).

*Case inclusion criteria*: (1) Meet the diagnostic criteria for MOH: Patients should meet the 2013 International Classification of Headache Disorders, 3rd edition (ICHD-3) [1]. The ICHD-3 included the following: (A) Headache occurring on ≥15 days/month in a patient with a pre-existing headache disorder, (B) Regular overuse for ≥3 months of one or more drugs that can be taken for acute and/or symptomatic treatment of headache, and (C) Not better accounted for by another ICHD-3 diagnosis; (2) Headache exacerbated during medication-overuse; (3) No history of drug withdrawal; (4) No obvious organic complications; (5) Meet the criteria of successful completion of drug withdrawal (headache completely disappears or the number of headache days reduces by 50 % per month); (6) Meet the MOH relapse diagnostic criteria (ICHD-IIR) amended by the International Headache Society in 2006. *Case exclusion criteria*: (1) Patients with other types of secondary headaches; (2) Patients who refuse to enroll in the follow-up investigation; (3) Patients with other diseases requiring long-term use of pain medications; (4) Patients with other serious chronic diseases; (5) Patients whose medical records are incomplete.

### Questionnaire content and method

All patients were surveyed using a uniform questionnaire (a database was created using Excel 2010). The survey contents included the following: (1) Name, gender, age, education, occupation, average annual household income, marital status, place of residence, history of heavy smoking and drinking, history of caffeine consumption, other drugs and substances addiction, family history of headache, family history of MOH, and family history of other drugs and substance abuse; (2) Time for overuse of drugs, headache frequency (days/month), degree of headache (assessed by “pain score caliper” using the visual analog scale [VAS] in which 0 = no pain, 10 = severe pain), and duration of each attack before drug withdrawal; (3) The primary headache type, primary headache course, primary headache frequency (days/month), duration of each primary headache attack, and degree of primary headache (VAS score); (4) The types of overused drugs, including a single use of non-steroidal anti-inflammatory drug (NSAID) analgesics, Chinese patent medicine, benzodiazepine compound analgesics or a combination of analgesics; (5) Frequency of drug use per month (days/month), headache frequency after drug withdrawal (days/month), duration of each attack after withdrawal, and degree of headache after withdrawal (VAS score); (6) Coexisting disorders such as anxiety, depression (diagnostic criteria for both is found in the American psychiatric diagnostic and statistical manual, 4th edition) [8], sleep disorders, and other discomfort; (7) Preventive medicine and the time taken for the application of preventive medicine. Investigators determined a patient’s relapse by outpatient appointment or telephone follow-up, according to the relapse criteria of the ICHD-IIR. Patients were divided into the case group and the control group. Investigations of the contents of the 38 qualifications were completed according to the original headache medical records and telephone follow-up of patients and were processed by a unified digital coding method.

### Statistical analysis

Count data (such as demographics) were analyzed by the chi-square test or Fisher’s exact test. The *t*-test was used to determine the normal distribution, and the Wilcoxon test was used to determine the skewed distribution of the data. For the multivariate logistic regression analysis, statistically significant (*P* < 0.05) variables were elected as independent variables and took a significance level of α = 0.05. All data were analyzed using Statistical Product and Service Solutions (SPSS) 17.0 software (IBM Company, Chicago, Illinois in the United State).

## Results

### General basic data analysis

Data from the 86 patients (aged 18–66 years; mean: 43.4 ± 10.8 years) revealed 13 (15.1 %) cases of MOH for males and 73 (84.9 %) cases for females. Overall, primary headache and tension-type headache occurred in 80 (93 %) and six (7 %) patients respectively. Overused acute medication, such as compound analgesics, occurred in 63 (73.3 %) patients. Twenty-three (26.7 %) patients took a single acute medication, including 16 individuals that used only NSAIDs (e.g. ibuprofen, acetaminophen, and aspirin) and six patients that only used proprietary Chinese medicine. One patient only used benzodiazepines, such as diazepam, to relieve pain.

After clinic interviews, 59 (68.6 %) patients who did not have medication overuse phenomenon were assigned as the control group. Based on the ICHD-IIR, 27 (31.4 %) patients were found to have MOH relapse and thus were assigned as the case group.

### Univariate analysis

#### Demographic and sociological indicators

Both the case and control group comprised 27 patients (31 to 65 years; mean: 46.6 years ± 10.8 years) and 59 patients (18 to 66 years; mean: 42.0 years ± 10.6 years) respectively. The data was consistent with a normal distribution. There was no statistical significance between the case and control group with respect to age, gender, education level and so on (Table 1).

**Table 1:** Demographic and sociological indicators (*n*)

	Indicators	Case group (*n* = 27)	Control group (*n* = 59)	*χ* ^2^	*P*
Gender	Male	6 (22.2 %)	7 (11.9 %)		0.330*
	Female	21 (77.8 %)	52 (88.1 %)		
Education level	Junior high school、primary school or lower	17 (63.0 %)	31 (52.5 %)	0.816	0.366
	Senior high school, university or higher	10 (37.0 %)	28 (47.5 %)		
Occupational status	Unemployed	6 (22.2 %)	6 (10.2 %)	5.371	0.068
	Student	0 (0 %)	8 (13.6 %)		
	Farmer	10 (37.0 %)	16 (27.1 %)		
	Worker	4 (14.8 %)	13 (22.0 %)		
	Cadre or other	7 (25.9 %)	16 (27.1 %)		
Marital status	Unmarried	0 (0 %)	10 (16.9 %)	5.941	0.051
	Married	24 (88.9 %)	45 (76.3 %)		
	Divorced or widowed	3 (11.1 %)	4 (6.8 %)		
Residence	Rural areas and suburbs	22 (81.5 %)	38 (64.4 %)	2.560	0.110
	City	5 (8.5 %)	21 (35.6 %)		
Household income	Less than 60,000/year	14 (51.9 %)	25 (42.4 %)	0.672	0.413
	More than 60,000/year	13 (48.1 %)	34 (57.6 %)		
Smoking history	No	16 (59.3 %)	47 (79.7 %)	4.345	0.114
	Less than 20/day	5 (18.5 %)	4 (6.8 %)		
	More than 20/day	6 (22.2 %)	8 (16.3 %)		
Drinking history(per day)	No	21 (77.78 %)	52 (88.14 %)		0.330*
	Yes	6 (22.22 %)	7 (11.86 %)		
History of caffeine intake	No	22 (81.48 %)	52 (88.14 %)		0.505*
	Yes	5 (18.52 %)	7 (11.86 %)		
Other drug history and substance addiction	No	21 (77.8 %)	50 (84.7 %)		0.542*
	Yes	6 (22.2 %)	9 (15.3 %)		
Family history of headache	No	12 (44.4 %)	23 (39.0 %)	0.229	0.632
	Yes	15 (55.6 %)	36(61.0 %)		
MOH family history	No	24 (88.9 %)	57 (96.6 %)		0.176*
	Yes	3 (11.1 %)	2 (3.4 %)		
Family history of addiction to other drugs and substances	No	22 (81.5 %)	52 (88.1 %)		0.505*
	Yes	5 (18.5 %)	7 (11.9 %)		

Figures in brackets as a percentage. *For Fisher’s exact test

#### MOH characteristics before drug withdrawal

Medication overuse over time of the case group was found to be significantly higher (*P* = 0.037) than that of the control group (Table 2). The headache frequency of the case group was markedly (*P* = 0.035) higher than that of the control group. There was no significant difference in the degree of headache pain episodes (*P* = 0.127) and duration of each attack (*t* = 1.646, *P* = 0.103) between the two groups.

**Table 2:** MOH characteristics before drug withdrawal

Indicators	Case group	Control group	Statistic	*P*
Medication overuse over time(year)	4.04 ± 1.126	3.37 ± 1.731	*t* = 2.124	0.037
Headache frequency (days/month)	24.07 ± 4.811	21.76 ± 4.573	*t* = 2.140	0.035
The degree of headache (VAS)	6 ~ 9 (8)	6 ~ 9 (8)	*Z* = −1.528	0.127
Duration of each attack (h)	10.15 ± 6.707	7.90 ± 5.473	*t* = 1.646	0.103

Normal distribution data with‾x ± s, statistics as the t value; Skewed distribution data with poor and median (in parentheses), statistics as the z value.

#### Characteristics of primary headache

From the 86 patients, 82 (95.4 %) and four (4.65 %) patients had primary headache that was migraine and tension-type headache respectively. From the 27 patients in the case group, 25 (92.6 %) and two (7.4 %) patients had primary headache that was migraine and tension-type headache respectively. From the 59 patients in the control group, 57 (96.6 %) and two (3.4 %) patients had primary headache that was migraine and tension-type headache. There was no statistical difference (*P* = 0.587) in the type of primary headache between the two groups. There was, however, a significant difference in the course of primary headache (*P* < 0.001) and primary headache frequency (*P* = 0.007) between the two groups. There was no significant difference between the two groups for the degree of primary headache (*P* = 0.058) and the duration of each episode (*P* = 0.055) (Table 3).

**Table 3:** Characteristics of primary headache

Indicators	Case group	Control group	Statistic	*P*
Course of primary headache	10 ~ 45 (20)	2 ~ 35 (8)	*Z* = −5.973	< 0.001
Headache frequency (days/month)	8.70 ± 2.072	7.12 ± 2.607	*t* = 2.780	0.007
Duration of each attack (h)	1 ~ 6 (3)	1 ~ 12 (2)	*Z* = −1.922	0.055
Headache degree (VAS)	6.22 ± 1.050	5.78 ± 0.966	*t* = 1.918	0.058

Normal distribution data with‾*x ± s*, statistics as the t value; Skewed distribution data with poor and median (in parentheses), statistics as the z value

#### Overuse of drugs

There was no significant difference in the overuse of drugs in the case group and the control group—whether only a single component of acute medication was used or whether a compound preparation or analgesic were used (Table 4).

**Table 4:** Overuse of drugs (*n*)

Indicators	Case group (*n* = 27)	Control group (*n* = 59)	*χ* ^2^	*P*
Use only a single component drug	9 (33.3 %)	14 (23.7 %)	0.872	0.350
Use only NSAID	6 (22.2 %)	10 (17.0 %)	0.340	0.560
Use only Chinese patent medicine	3 (11.1 %)	3 (5.1 %)		0.373*
Use only benzodiazepines	0	1 (1.7 %)		1.000*
Combination of compound preparations or painkillers	18 (66.7 %)	45 (76.23 %)	0.872	0.350

Figures in brackets as a percentage. *For Fisher’s exact test

#### Drug use and withdrawal situation

Table 5 showed that there was no significant difference in the frequency of acute medication use (*P* = 0.051) and the duration of each episode (*P* = 0.270) between the case group and the control group. After drug withdrawal, headache frequency was significantly higher in the case group (*P* < 0.001), headache degree was also higher than the control group (*P* = 0.024).

**Table 5:** Drug use and withdrawal situation

Indicators	Case group	Control group	Statistic	*P*
Drug use frequency (days/month)	24.26 ± 5.035	22.12 ± 4.521	*t* = 1.982	0.051
Headache frequency After drug withdrawal (days/month)	7.67 ± 1.961	3.42 ± 1.499	*t* = 11.026	< 0.001
Duration of each attack (h)	1 ~ 4 (2)	1 ~ 6 (2)	*Z* = −1.104	0.270
Headache degree (VAS) after drug withdrawal	5.52 ± 1.341	4.85 ± 0.943	*t* = 2.348	0.024

Normal distribution data with‾*x ± s*, statistics as the t value; Skewed distribution data with poor and median (in parentheses), statistics as the z value

#### Comorbidity after drug withdrawal

There was no significant difference between the case group and the control group for comorbidity after drug withdrawal, including sleep disorders (*P* = 0.139), anxiety (*P* = 0.254), depression (*P* = 1.000) and other discomfort (*P* = 0.221) (Table 6).

**Table 6:** Comorbidity after drug withdrawal (*n*)

Indicators	Case group (*n* = 27)	Control group (*n* = 59)	*χ* ^2^	*P*
Sleep disorders	17 (62.96 %)	27(45.76 %)	2.193	0.139
Anxiety	8 (29.63 %)	11(18.64 %)	1.299	0.254
Depression	0 (0 %)	1 (1.70 %)		1.000*
Other symptoms	7 (25.9 %)	8 (13.6 %)		0.221*

Figures in brackets as a percentage. *For Fisher’s exact test

#### Prevention of drug use

All 86 patients underwent drug treatment to prevent headache after drug withdrawal. There was a significant difference (*P* = 0.009) in the timeframe for the application of drug prevention between the two groups (Table 7).

**Table 7:** Prevention of drug use

Application time of prevention drug	Case group (*n* = 27)	Control group (n = 59)	*χ* ^2^	*P*
< 6 months	11 (40.74 %)	9 (15.25 %)	6.742	0.009
> 6 months	16 (59.26 %)	50 (84.75 %)		

#### Risk factors for preliminary screening

Seven risk factors were screened through the analysis of the 31 factors (such as age; *P* < 0.05), including medication overuse over time, headache frequency before drug withdrawal, duration of primary headache, primary headache frequency, headache frequency after drug withdrawal, severity of headache after drug withdrawal, and the timeframe for the application of drug prevention (Table 8).

**Table 8:** Risk factors for preliminary screening

Indicators	Statistics	*P*
Medication overuse time	*t* = 2.124	0.037
Headache frequency before drug withdrawal	*t* = 2.140	0.035
Duration of primary headache	*Z* = −5.973	< 0.001
Primary headache frequency	*t* = 2.780	0.007
Headache frequency after drug withdrawal	*t* = 11.026	< 0.001
Severity of headache after drug withdrawal	*t* = 2.348	0.024
Application time of prevention drug	*χ* ^2^ = 6.742	0.009

### Multivariate analysis

Multi-factor unconditional logistic regression analysis was carried on medication overuse over time, headache frequency before drug withdrawal, duration of primary headache, primary headache frequency, headache frequency after drug withdrawal, severity of headache after drug withdrawal, and the timeframe for the application of drug prevention. Three indicators (headache frequency before drug withdrawal, duration of primary headache, and headache frequency after drug withdrawal) were applied to the regression equation and were found to be independent risk factors for MOH relapse (Table 9).

**Table 9:** Multi-factor unconditioned Logistic regression analysis results

Name of factors	B	S.E.	Wals	Df	Sig.	Exp (B)	EXP(B) 的 95 % C.I.	
							lower limit	upper limit
Years of medication overuse	1.541	1.055	2.131	1	0.144	4.669	0.590	36.953
Headache frequency before drug withdrawal	0.559	0.271	4.237	1	0.040	1.748	1.027	2.976
Frequency of Primary headache	0.543	0.511	1.130	1	0.288	1.721	0.633	4.683
Duration of primary headache	0.226	0.114	3.930	1	0.047	1.254	1.003	1.567
Headache frequency after drug withdrawal	3.078	1.351	5.192	1	0.023	21.721	1.538	306.780
Severity of headache after drug withdrawal	-.830	1.002	0.686	1	0.407	0.436	0.061	3.107
Application time of prevention drug	−1.445	1.591	0.825	1	0.364	0.236	0.010	5.331
Constant	−38.412	19.384	3.927	1	0.048	0.		

## Discussion

A recent number of international clinical studies on the long-term prognosis of MOH have revealed that MOH has a high relapse rate, with 30–45 % of MOH patients relapsing after withdrawal and a 1-year relapse rate of 22–44 % [7]. In some large-scale clinical follow-ups of a 6-year long-term prognosis, patients experience a relapse rate of 24–43 %, with the majority of relapse cases occurring within a year after withdrawal [9]. Despite these findings, no such research has been undertaken in China and thus, MOH relapse rate and independent risk factors remain unknown for this country.

The present study involved a total of 86 MOH patients with complete data and who met the conditions, and were followed up 2 months after withdrawal. Our findings showed no significant difference between two groups regarding demographic and sociological characteristics. These results corroborate those of Zidverc-Trajkovic et al. [10, 11]. However, a study by Sances et al. [12] believes that heavy smoking and drinking may increase the relapse rate in the first year, most likely due to the effect of psychoactive substances (such as alcohol, tobacco, and opium) in patients with addictive substance abuse. Patients with a high risk of suffering from MOH are more susceptible to drug dependence and are more likely to relapse after withdrawal [13].

In the present study, medication overuse overtime and headache frequency in the case group were significantly higher than those of the control group when examining MOH characteristics before drug withdrawal. When the regression equation was applied to headache frequency before drug withdrawal, it was found to be an independent risk factor for MOH relapse. Our results indicated that the higher the headache frequency prior to drug withdrawal, the higher the relapse after drug withdrawal.

A significant difference was found between the two groups for primary headache and primary headache frequency. The regression equation analysis revealed that primary headache frequency was another independent risk factor for MOH relapse and that an increase in relapse was associated with high-frequency primary headache. These findings are consistent with those of Sances et al. [12] who has shown that primary headache frequency is an independent risk factor that affects the outcome of MOH treatment.

Our results also showed that the overuse of drugs was not significantly different between the two groups. Studies have shown that the MOH relapse rate is the lowest with acute use of ergot amines, and triptans drugs have a significantly lower (21 %) effect than analgesic drugs (71 %), composite analgesics, and barbiturates (e.g. butalbital) [14]. Opioids are also most likely to cause MOH relapse [11].

Headache frequency and headache degree were significantly different between the two groups after drug withdrawal. The MOH regression analysis for headache frequency revealed that it was the most important independent risk factor to affect relapse, thereby indicating that the higher the headache frequency after drug withdrawal, the more likely that the MOH patient will relapse. Rossi et al. [11] have shown that patients with high frequency of headache after withdrawal are at higher risk of MOH relapse. Other studies have confirmed that after a successful drug withdrawal and a 3-month drug prevention treatment, 60 % of the chronic migraine patients with headache frequency greater than 10 days/month re-develop MOH [15].

After drug withdrawal, there was no significant difference in MOH comorbidity between the two groups. There was a significant difference in the timeframe for the application of drug prevention drug between two groups, but the MOH regression equation was not used for this index. The purpose of preventive treatment was to reduce the frequency of headache to decrease the frequency of taking drugs. Reducing headache frequency may decrease mood swings during headache as well as reduce the early use of medication prevent headache. As MOH relapse rate is high, preventive treatment is best carried out long-term.

Headache frequency of drug withdrawal, duration of primary headache, and headache frequency after drug withdrawal were the three factors screened out to be independent risk factors for MOH relapse in the follow-up of the 86 patients. However, there were some limitations of this study: (1) The sample size was small, (2) patients who were diagnosed with short-term (every 3 months/time) MOH were not followed up continuously, and (3) dynamic tracking was not applied. Therefore, there may have been some bias regarding the return visit data, and this should be avoided in future studies.

To prevent MOH relapse, drug dependence screening using questionnaires in patients with primary headache in clinical routing becomes particularly important. Clinicians find that the patients’ tendency for drug dependence prevents the occurrence of MOH. In the 2011 International Headache Conference, the majority of experts agreed that MOH patients should discontinue drug overuse (withdrawal treatment) [16]. Preventing the overuse of drugs can relieve headaches or completely eradicate them, as well as enhance the efficacy of preventive drugs. Because withdrawal reactions may occur after the withdrawal of certain drugs—particularly benzodiazepines—and other drugs such as opiates and barbiturates, patients taking these drugs should withdraw from them slowly and gradually. Topiramate and a local injection of onabotulinumtoxinA have been shown to exhibit the same efficacy as therapeutic agents for re-prophylaxis after detoxification in chronic migraine patients with or without medication overuse [17, 18]. Grande et al. [19] have shown that cognitive behavior therapy (psychotherapy combined with drug therapy) is more effective in improving headache and the quality of life of patients. Prophylactic pharmacologic measures as well as psychological support, education, and surveillance to prevent relapses are considered to enhance the chances of success in MOH relapse [20]. In recent years, MOH patients have had a high risk of relapse, and this relapse rate is too high. Therefore, clinicians need to develop effective relapse prevention strategies to effectively reduce the MOH relapse rate.

## Conclusion

MOH relapse in China may be related to three risk factors: headache frequency before drug withdrawal, duration of primary headache, and headache frequency after drug withdrawal.
